# Effects of human serum albumin on post-mortem changes of malathion

**DOI:** 10.1038/s41598-021-91145-y

**Published:** 2021-06-02

**Authors:** Yoshikazu Yamagishi, Hirotaro Iwase, Yasumitsu Ogra

**Affiliations:** 1 Laboratory of Toxicology and Environmental Health, Graduate School of Pharmaceutical Sciences, Chiba University, Chuo, Chiba 260-8675 Japan; 2 Department of Legal Medicine, Graduate School of Medicine, Chiba University, Chuo, Chiba 260-8670 Japan

**Keywords:** Chemical biology, Health care

## Abstract

Malathion, diethyl 2-[(dimethoxyphosphinothioyl)thio]butanedioate, is one of most widely used organophosphoryl pesticide, and it has been detected in several clinical cases of accidental exposure and suicide. It is reported that the observed malathion concentration in blood of persons who suffer from malathion poisoning is smaller than the expected concentration. Because malathion is bound to human serum albumin (HSA), recovery of malathion in the free form is insufficient. We detected malathion adducts in HSA by liquid chromatography quadrupole time-of-flight mass spectrometry (LC-Q/TOF–MS). The mass spectra showed that malathion was preferably bound to the lysine (K) and cysteinylproline (CP) residues of HSA. The K- and CP-adducts of malathion were increased in vitro with a dose-dependent fashion when its concentration was smaller than the lethal dose. Further, the K-adduct was also detected in post-mortem blood of an autopsied subject suffering from intentional malathion ingestion. These results suggest that the K-adduct seems to be available to use a biomarker of malathion poisoning, and the determination of the K-adduct could make possible to estimate the amount of malathion ingestion.

## Introduction

Malathion, diethyl 2-[(dimethoxyphosphinothioyl)thio]butanedioate or *S*-1,2-bis(ethoxycarbonyl)ethyl *O,O*-dimethyl phosphorodithioate, is a broad-spectrum organophosphoryl pesticide marketed around the world. Malathion consists of an *O*,*O*-dimethylthiophospho moiety and a thiobutanedioate moiety linked by a phosphorus-sulfur bond, and gives an insecticidal effect by inhibiting acetylcholine esterase (AChE). This structural feature is common among many pesticides having the same toxicity^1^. The inhibition of AChE by malathion leads to the accumulation of acetylcholine (ACh) in the synaptic cleft, and is due to the high acute toxicity of malathion even in human and animals. Malathion is the fifth leading compound contributing to all poisoning deaths including accidents and suicide by pesticide ingestion in Japan^2^, and the lethal concentration of malathion in blood is 0.6–19 μg/mL^3^. Although the lethal concentration of malathion is sufficient for detection by instrumental analyses, malathion per se was not detected in post-mortem blood from malathion-poisoning subject^4^. In the context of forensic toxicology, the accurate determination of malathion concentration is compulsory to judge malathion poisoning death. However, there is no scientific evidence to reasonably explain why malathion concentration in post-mortem blood cannot be accurately determined.

Two mechanisms are speculated to explain alterations of malathion concentration in blood after death. The first mechanism is that malathion may be decomposed by the esterase activity of human serum albumin (HSA). HSA is the most abundant protein in plasma and serves as a carrier protein for many endogenous and exogenous compounds in bloodstream^5^. On the other hand, malathion is decomposed into malathion monoacid by carboxylesterase, and malathion monoacid is the major metabolite detected in human^6^. Thus, it is speculated that HSA acts as a carboxylesterase in post-mortem blood. Indeed, esterase activity of HSA is reported^7^. Malathion becomes a substrate of esterase, and may be decomposed in blood by HSA even though the esterase activity of HSA is relatively low. The second mechanism is that malathion may be bound to HSA and/or hemoglobin (Hb). Several blood proteins, including HSA and Hb, can bind malathion. It was reported that organophosphorus compounds having the same structural characteristics as malathion are bound to these proteins^8–10^. Therefore, we intended to evaluate the speculated mechanisms mentioned above, i.e., the esterase activity of HSA and the interaction of malathion with blood proteins. To this end, we measured malathion and its metabolites in HSA and Hb solutions. We also evaluated malathion bound to blood protein(s). In this study, liquid chromatography quadrupole time-of-flight mass spectrometry (LC-Q/TOF–MS) was used to meet the analytical requirements for these evaluations. LC-Q/TOF–MS is an effective technique to evaluate unidentified metabolites and to identify protein adducts because it can provide the accurate molecular mass by TOF–MS and molecular information by tandem MS^11^.

## Experimental

### Ethics approval

This study was performed with approval from The Research Ethics Committee of Graduate School of Pharmaceutical Sciences (Approval No. R001) and School of Medicine (Approval No. 2819), Chiba University. Use of autopsy samples for analyses was carried out in accordance with The Code of Ethics of the World Medical Association (Declaration of Helsinki). We have included a disclaimer on our official website stating that we occasionally collect samples from autopsies for research use. Families who do not consent to it can freely opt out by informing us. We received the freely-given informed consent of healthy volunteers in writing.

### Chemicals

Acetonitrile (ACN, LC/MS grade), distilled water (LC/MS grade), formic acid (FA, 98–100%), and 1 mol/L ammonium formate solution (HPLC grade) were purchased from Kanto Chemical (Tokyo, Japan). Tris–HCl buffer (0.5 mol/L) was gained from Corning (MA, USA). Diazepam-d5 was purchased from Hayashi Pure Chemical Ind., Ltd. (Osaka, Japan). Malathion (TraceSure®), trifluoroacetic acid (TFA, HPLC grade), and glutathione (GSH, special grade) were obtained from Fujifilm Wako Pure Chemical (Osaka, Japan). HSA, Hb, pronase, and Amicon ultra-0.5 centrifugal filter units with molecular weight cut-off of 10 kDa were purchased from Sigma-Aldrich (St. Louis, MO, USA).

### Instruments

Two liquid chromatographs hyphenated with quadrupole/time-of-flight mass spectrometers were used. X500R (AB Sciex, Foster City, CA, USA) and 5600 + QTOF (AB Sciex) were equipped with Prominence UFLC (Shimadzu, Kyoto, Japan) and Nexera X2 (Shimadzu) as their liquid chromatographs, respectively. The conditions for liquid chromatography and mass spectrometry were according to our previous reports^11^, and summarized in Table 1.

**Table 1:** Liquid chromatography and mass spectrometry conditions.

	X500R	5600 + QTOF^a^
**LC conditions**		
LC system	Prominence UFLC (Shimadzu, Kyoto, Japan)	Nexera X2 (Shimadzu)
Column	Atlantis T3 C18 column, 50 × 2.0 mm I.D., 3 μm (Waters, Milford, MA, USA)	←
Injection volume (µL)	2	20
Total flow (mL/min)	0.3	0.2
Oven (ºC)	40	←
Elution buffer A	0.1% Formic acid and 10 mM ammonium formate	←
Elution buffer B	Acetonitrile (ACN)	←
Gradient curve (A/B)	90/10 (0 min)–40/60 (12 min) – 0/100 (18–23 min)–90/10 (23–30 min)	←
**MS conditions**^**b**^		
Polarity	Positive	←
Mode	IDA	Product ion scan
*m/z* range	50–800	50–400
Accumulation time (ms)	100	←
ISVF (V)	5500	←
DP (V)	60	←
CE (V)	20	←
CES (V)	5	←
IRD (ms)	67	←
IRW (ms)	25	←
GS1 (psi)	50	←
GS2 (psi)	80	←
CUR (psi)	25	←
TEM (°C)	500	350

^a^ ← , The conditions were identical to those used in X500R.

^b^*ISVF* ion spray voltage floating, *DP* declustering potential, *CE* collision energy, *CES* collision energy spread, *IRD* ion release delay, *IRW* ion release width, *GS1* source gas 1, *GS2* source gas 2, *CUR* curtain gas, *TEM* ion source temperature.

### Recovery of malathion from reaction mixture with HSA and Hb

A 0.1 mL of a reaction mixture including blood proteins (final concentrations, HSA 40 mg/mL or Hb 100 mg/mL) and malathion (final concentration, 1 μg/mL) was incubated at 37 °C for 0 and 24 h. We used HSA and Hb at the concentrations reported in the previous literatures^8,11^. After the incubation, diazepam-d5 (50 ng/mL) as the internal standard in 1.9 mL of ACN was added to the reaction mixture. The mixture was vortexed, sonicated, and centrifuged at 9982×*g* for 10 min, and the supernatant was injected to LC-Q/TOF–MS (X500R) to determine malathion concentration and detect malathion monoacid.

For the evaluation of the time-dependent recovery of malathion from HSA, a 0.1 mL of a reaction mixture containing 40 mg/mL HSA and malathion (final concentration, 1 μg/mL) was incubated at 37 °C for 0, 1, 2, 4, 8, and 24 h. After the incubation, diazepam-d5 (50 ng/mL) as the internal standard in 1.9 mL of ACN was added to the reaction mixture. The mixture was vortexed, sonicated, and centrifuged at 9982×*g* for 10 min, and the supernatant was injected to LC-Q/TOF–MS (X500R) to determine malathion concentration.

### Detection, identification, and validation of malathion adducts in HSA

A 0.11 mL aliquot of a reaction mixture containing 40 mg/mL HSA and 10 mg/mL malathion in 0.5 M Tris–HCl buffer was incubated at 37 °C for 24 h. The enzyme digestion by pronase was performed by the methods reported in our previous literature^11^. Briefly, a 6 mg portion of pronase was added to the reaction mixture, and an overnight incubation was carried out at 37 °C. To detect and identify malathion adducts in Hb peptides, 0.3 mL of 0.5% TFA was added to 0.1 mL of the reaction mixture after the second incubation. The reaction mixture was vortexed and centrifuged at 9982×*g* for 10 min, and the supernatant was injected to LC-Q/TOF–MS (X500R).

For the validation of malathion adducts in HSA peptides, a 0.5 mL of a reaction mixture consisting 40 mg/mL HSA and 1, 3, 10, 30, 100, 300 or 1000 μg/mL malathion in 0.5 M Tris–HCl buffer was incubated at 37 °C for 24 h. After the incubation, 0.5 mL of the reaction mixture was transferred into an ultrafiltration device (Amicon ultra-0.5 centrifugal filter unit) and centrifuged at 9984×*g* for 10 min. The aliquot was washed three times with 0.3 mL of 0.5 M Tris–HCl buffer and centrifuged again at 9984×*g* for 10 min. The enzyme digestion by pronase was performed by the methods reported in our previous literature^11^. Briefly, a 30 mg portion of pronase was added to the reaction mixture, and an overnight incubation was carried out at 37 °C. A 0.3 mL aliquot of 0.5% TFA was added to a 0.1 mL portion of the reaction mixture after the second incubation. The mixture was vortexed and centrifuged at 9982×*g* for 3 min, and the supernatant was subjected to LC-Q/TOF–MS (5600 + QTOF). Precursor ions of the lysine (K)-adduct and the cysteinylproline (CP)-adduct of malathion moiety were detected at *m/z* 271.1 and *m/z* 395.1, respectively. Product ions of the K-adduct and the CP-adduct were detected at *m/z* 271.0876 and *m/z* 217.0641, respectively.

### Detection of malathion in post-mortem samples from malathion poisoning subject and K- and CP-adducts in blood sample from malathion poisoning subject

Femoral vein blood, heart blood, urine, and stomach content were collected from a 72-year-old woman approximately 72–168 h after death by intentional ingestion of malathion-containing pesticide. Peripheral blood was collected from healthy volunteers to serve as control.

A 0.5 mL of the blood sample was transferred into an Amicon ultra-0.5 centrifugal filter unit and centrifuged at 9984×*g* for 10 min. The aliquot was washed three times with 0.3 mL of 0.5 M Tris–HCl buffer and centrifuged again at 9984×*g* for 10 min. The enzyme digestion by pronase was performed by the methods reported in our previous literature^11^. Briefly, 30 mg portion of pronase and 0.3 mL of 0.5 M Tris–HCl buffer were added to the aliquot of the blood sample, and the blood sample was incubated at 37 °C overnight. After centrifugation at 9984×*g* for 10 min, 0.3 mL of 0.5% TFA was added to a 0.1 mL portion of the blood sample after the incubation. The reaction mixture was centrifuged at 9982×*g* for 3 min, and the supernatant was injected to LC-Q/TOF–MS (5600 + QTOF). Precursor ion of the K-adduct and the CP-adduct of malathion moiety were detected at *m/z* 271.1 and *m/z* 395.1, respectively. Product ions of the K-adduct and the CP-adduct were detected at *m/z* 271.0876 and *m/z* 217.0641, respectively.

### Statistics

Data are expressed as a means ± standard deviation (SD). The Student’s *t*-test and Tukey's test were performed for comparisons between two groups and among all groups, respectively. Asterisks (* and **) denote significance at *p* < 0.05 and *p* < 0.01, respectively.

## Results

### Stability of malathion in solution

**Figure 1:**
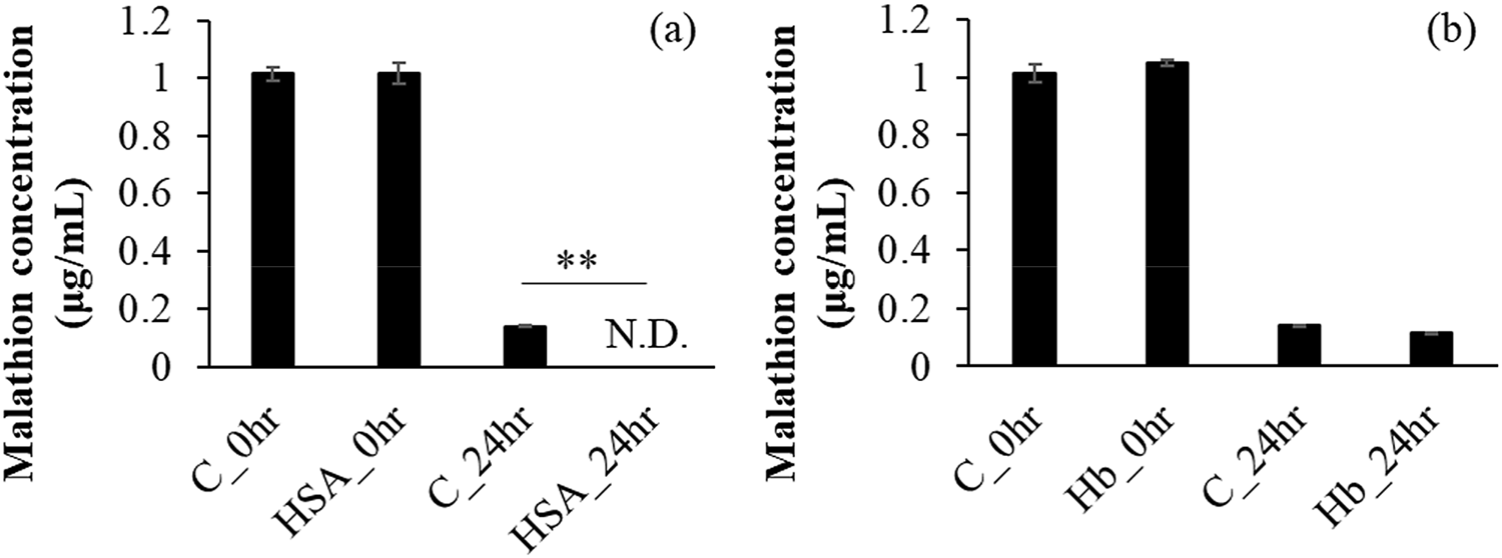
Effect of blood proteins on malathion concentration. One μg/mL malathion was incubated in HSA solution (**a**) or Hb solution (**b**). Tukey’s test was performed for comparison between groups. Double asterisks (**) indicate significant difference at *p* < 0.01.

Malathion concentration that decreased after 24 h incubation in 0.5 M Tris–HCl buffer served as control (Fig. 1). The percentage decrease of malathion concentration in 0.5 M Tris–HCl buffer, HSA solution, and Hb solution at 24 h compared with that at 0 h was 86.0%, 100%, and 89.2%, respectively. A significant difference in malathion concentration at 24 h was noted between control and HSA solution, but not between control and Hb solution. Furthermore, a signal at the retention time of 8.4 min was detected at *m/z* corresponding to malathion monoacid in control and Hb solution measured by TOF–MS, but the signal at the retention time of 8.4 min was not detected in the HSA solution (Fig. S1).

**Figure 2:**
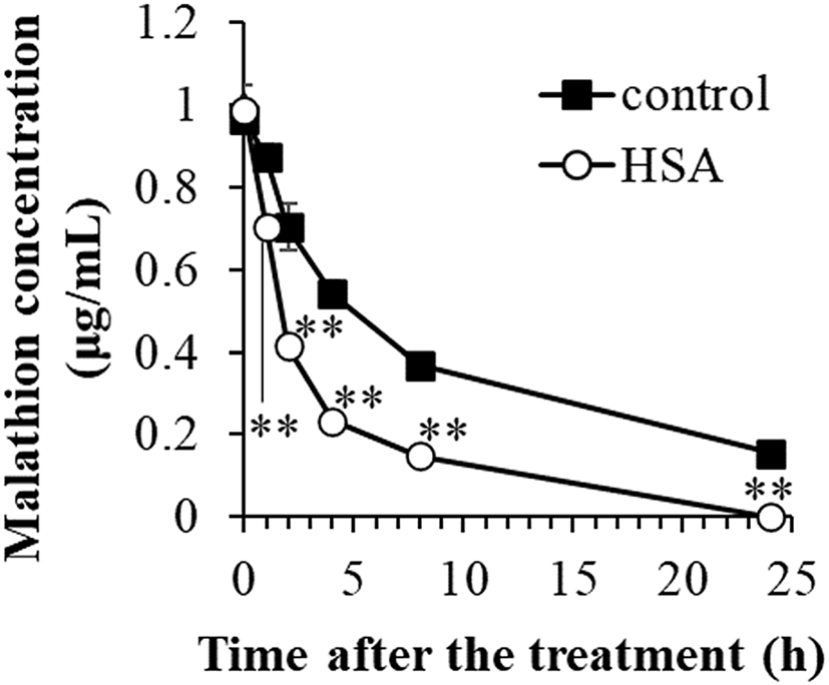
Time course of malathion concentration in HSA solution. One μg/mL malathion was incubated in HAS solution. Student’s t-test was performed for comparison between control and HSA. Double asterisks (**) indicate significant difference at *p* < 0.01.

Malathion concentrations in control and HSA solution showed a time-dependent decrease, and and a significant difference in concentration between the two groups was noted 1 h after the incubation (Fig. 2).

### Detection and identification of malathion adducts in HSA peptides

**Figure 3:**
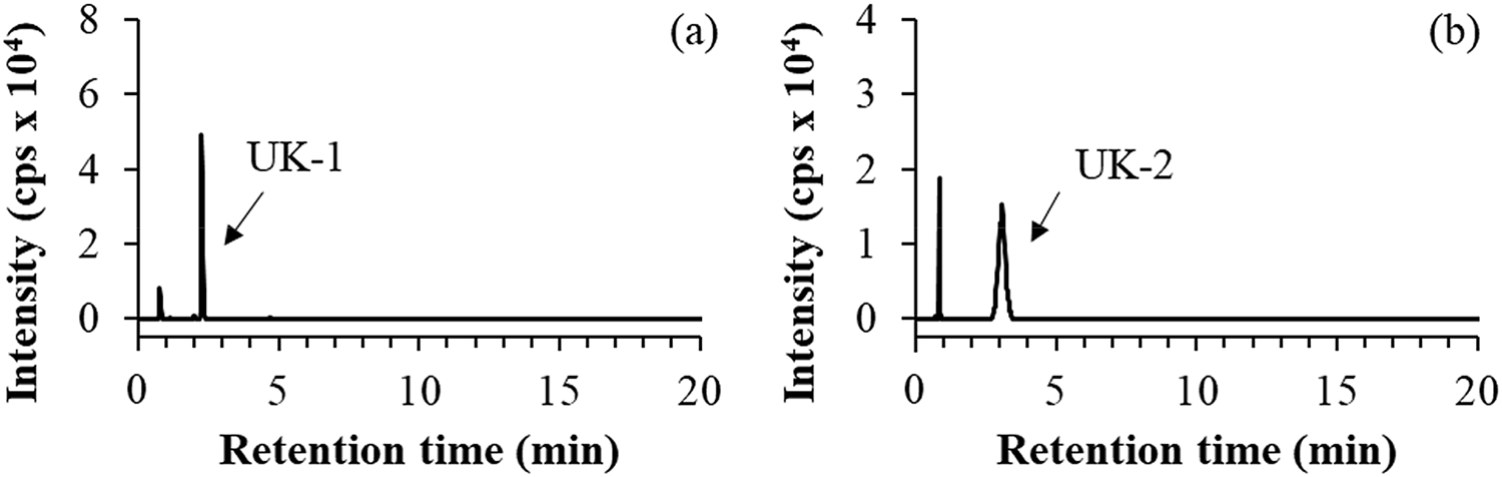
Elution profiles at *m/z* 271.0881 (**a**) and 395.0947 (**b**) in pronase-digested HSA with malathion treatment. Retention times of UK-1 and UK-2 were 2.3 and 3.1, respectively.

The difference analysis of the mass spectra of enzyme-digested HSA peptides with and without malathion treatment was performed. Two specific ions detected only in the malathion-treated HSA were extracted at *m/z* 271.0881 and 395.0947. The one at *m/z* 271.0881 eluted at the retention time of 2.3 min and was named UK-1 (Fig. 3a). The other ion at *m/z* 395.0947 eluted at the retention time of 3.1 min and was named UK-2 (Fig. 3b). The peak appearing before 1 min seemed to be a non-specific signal and was therefore not considered.

**Figure 4:**
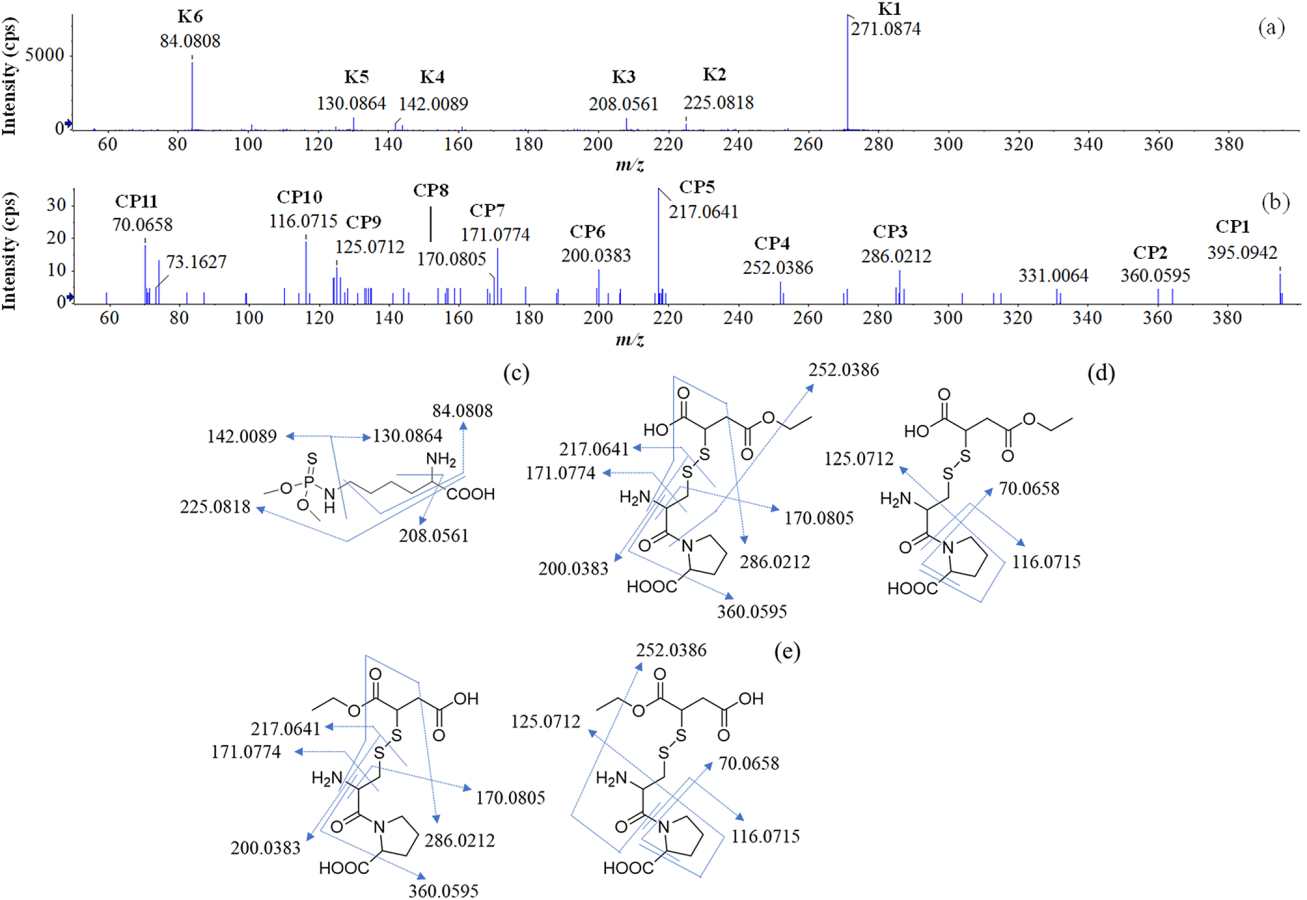
MS/MS spectra of unknown malathion adducts detected in the positive ion mode. Unknown peaks UK-1 (**a**) and UK-2 (**b**) detected in Fig. 3 were subjected to LC-Q/TOF–MS analysis. The UK-1 (**c**) and UK-2 (**d**,**e**) ions were assigned. Two possible structures of UK-2 were proposed (**d**,**e**).

The results of MS/MS analyses of UK-1 and UK-2 are shown in Fig. 4a,b, respectively. In Fig. 4a, six ions were detected; the largest one was the precursor ion (UK-1) because its *m/z* was 271.0874, and was renamed K1. Five fragment ions were detected at *m/z* 225.0818, 208.0561, 142.0089, 130.0864, and 84.0808, and were named K2–K6 in decreasing order of size. The assignments of these ions are sumarized in Table 2. The Δ*m/z* values for K1–K6 were less than ± 2.4 ppm. These assignments unambiguously suggested that K-1 (UK-1) was *O*,*O*-dimethylthiophospholysine, named K-adduct (Fig. 4c). In Fig. 4b, one precursor and ten fragment ions were detected. The ion detected at *m/z* 395.0942 was the precursor ion (UK-2), and was renamed CP1. Ten fragment ions were detected at *m/z* 360.0595, 286.0212, 252.0386, 217.0641, 200.0383, 171.0774, 170.0805, 125.0712, 116.0715 and 70.0658, and were named CP2–CP11 in decreasing order of size. The assignments of these ions are sumarized in Table 3. These assignments suggested that CP-1 (UK-2) was *S*-((1-carboxy-3-ethoxy-3-oxopropyl)thio)cysteinylproline or *S*-((3-carboxy-1-ethoxy-1-oxopropan-2-yl)thio)cysteinylproline, named CP-adduct. Because the hydrolysis site of the diethyl succinate moiety of malathion could not be specified by the fragment ions, two possible structures were considered (Fig. 4d,e). Figure 4d shows the assignment for the fragment ions of one of the presumed CP-adducts.

**Table 2:** Assignment of precursor and product ions of K-adduct.

Peak label	Elemental composition	*m/z* theoretical	*m/z* measured	Δ *m/z* (ppm)
K1	C_8_H_20_N_2_O_4_PS	271.0876	271.0874	− 0.7
K2	C_7_H_18_N_2_O_2_PS	225.0821	225.0818	− 1.3
K3	C_7_H_15_NO_2_PS	208.0556	208.0561	2.4
K4	C_2_H_9_NO_2_PS	142.0086	142.0089	2.1
K5	C_2_H_8_NOPS	130.0863	130.0864	0.8
K6	C_5_H_10_N	84.0808	84.0808	0.0

**Table 3:** Assignment of precursor and product ions of CP-adduct.

Peak label	Elemental composition	*m/z* theoretical	*m/z* measured	Δ *m/z* (ppm)
CP1	C_14_H_22_N_2_O_7_S_2_	395.0941	395.0942	0.3
CP2	C_14_H_18_NO_6_S_2_	360.0570	360.0595	6.9
CP3	C_11_H_12_NO_4_S_2_	286.0202	286.0212	3.5
CP4	C_8_H_14_NO_4_S_2_	252.0359	252.0386	10.7
CP5	C_8_H_13_N_2_O_3_S	217.0641	217.0641	0.0
CP6	C_8_H_10_NO_3_S	200.0376	200.0383	3.5
CP7	C_7_H_11_N_2_O_3_S	171.0764	171.0774	5.8
CP8	C_8_H_12_NO_3_	170.0812	170.0805	− 4.1
CP9	C_6_H_9_N_2_O	125.0709	125.0712	2.4
CP10	C_5_H_10_NO_2_	116.0706	116.0715	7.8
CP11	C_4_H_8_N	70.0651	70.0658	10.0

**Figure 5:**
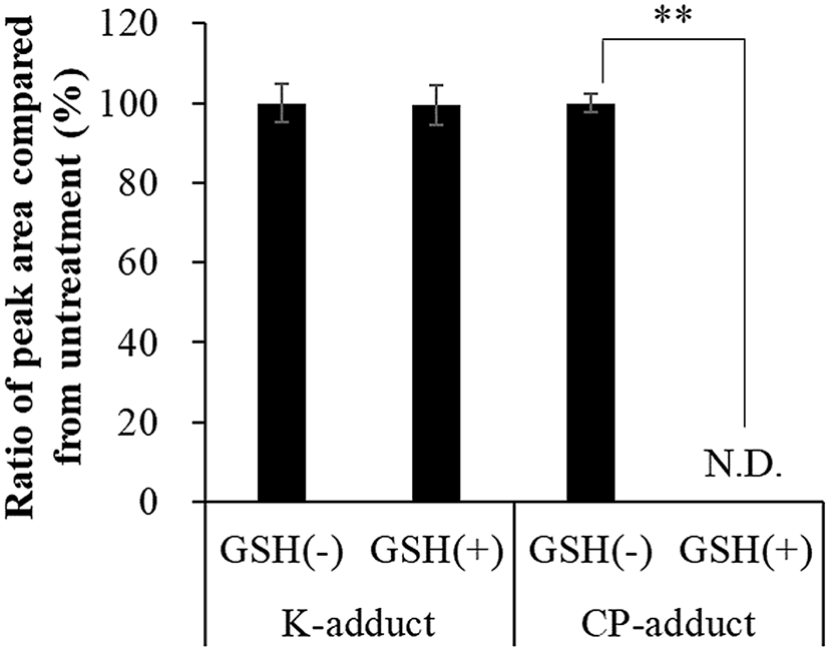
Effect of GSH on peak areas of K-adduct and CP-adduct in HSA. Ten μg/mL malathion and HSA were incubated without or with GSH. Student’s t-test was performed for comparison between without and with GSH treatment. Double asterisks (**) indicate significant difference at *p* < 0.01.

The CP-adduct, but not the K-adduct, was significantly decreased by GSH treatment for 24 h (Fig. 5). This suggests that the CP-adduct has a disulfide bond in its molecule, as indicated in Fig. 4d,e.

### Validation of LC-Q/TOF–MS and detection of malathion in post-mortem samples from malathion poisoning subject and K- and CP-adducts in blood sample from malathion poisoning subject

The K- and CP-adducts were detectable at 1.0 and 3.0 μg/mL or higher concentrations, respectively (Fig. S2). The relationship between malathion concentration and the peak areas of the K- and CP-adducts indicated good linearity, i.e., the correlation coefficients (r^2^) were 0.995 and 0.992, respectively.

Malathion concentrations in heart blood, urine, and stomach content collected from the autopsied subject who died of intentional malathion ingestion were 0.019, 0.079 and 8562 μg/mL, respectively. Malathion was not detected in femoral vein blood (Table 4).

**Table 4:** Malathion concentrations in autopsied subject who died of intentional malathion poisoning (μg/mL).

Case no	Femoral vein blood	Heart blood	Urine	Stomach content
A	N.D	0.019	0.079	8562

*N.D.* not detected.

**Figure 6:**
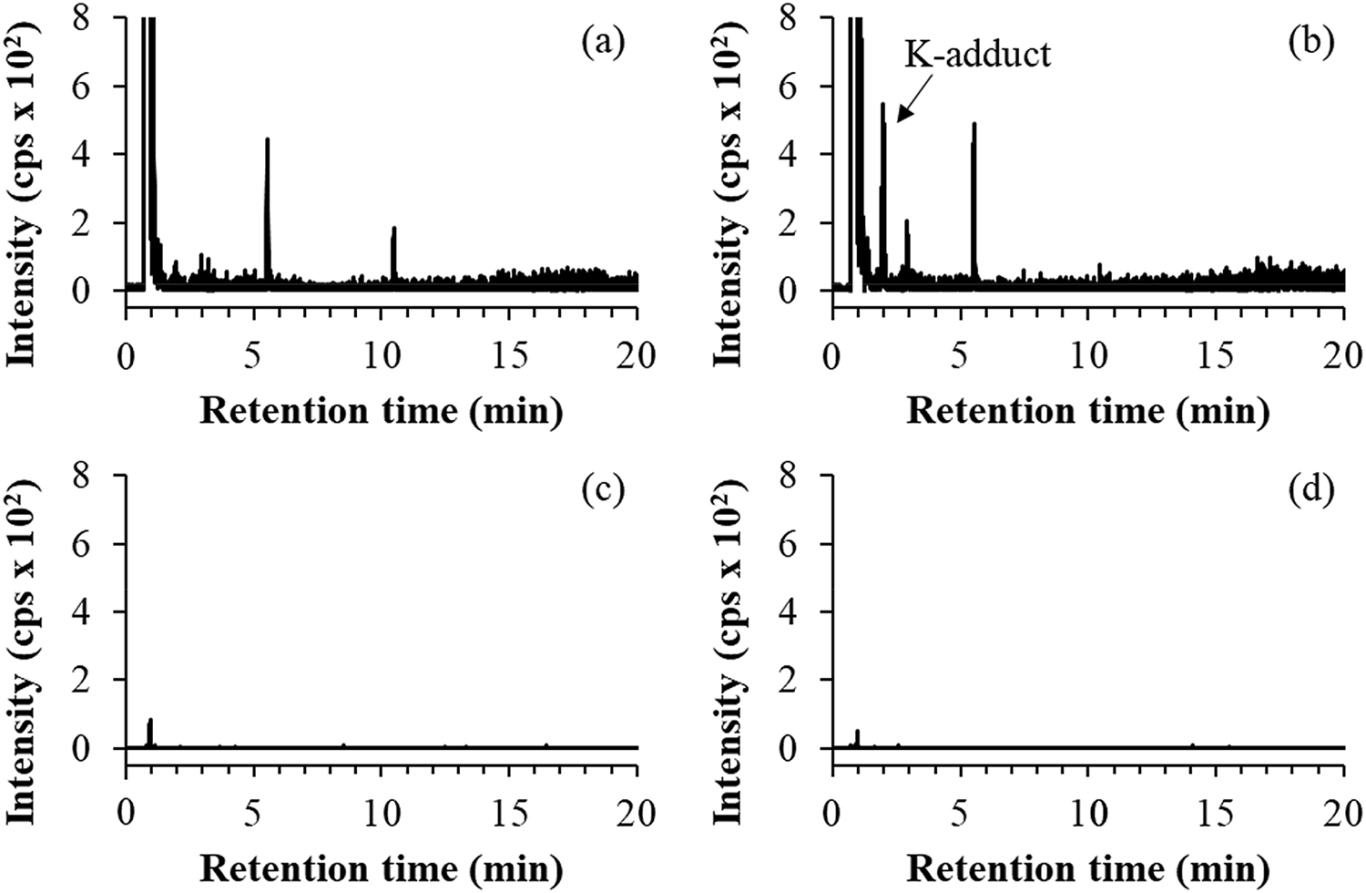
Elution profiles of K-adduct and CP-adduct in pronase-digested HSA from control (**a**,**c**) and malathion-poisoning subject (**b**,**d**).

Neither K- nor CP-adduct was detected in blood of a healthy volunteer (Fig. 6a,c). On the other hand, the K- adduct was specifically detected in blood collected from the autopsied subject who died of intentional malathion ingestion (Fig. 6b). However, no CP-adduct was detected in the same blood sample (Fig. 6d). Malathion concentration in this blood sample was estimated to be 9.5 μg/mL on the basis of the amount of K-adduct in blood.

## Discussion

One of primary factors for post-mortem decomposition of ester compounds are known to be plasma and bacterial esterases^12^. Because human hepatic carboxylesterases induced the decomposition of malathion^6^, we initially speculated that malathion was decomposed by HSA esterase activity. However, we detected no *m/z* signal corresponding to malathion monoacid, which is the degradation product of malathion by esterase. On the other hand, we found another mechanism by which HSA is implicated in the post-mortem decomposition of malathion, namely, the direct interaction of malathion with amino acid residues in HSA. HSA is the most abundant protein in blood plasma and thus, would probably play a critical role in the disappearance of malathion in blood.

It is reported that the phosphoryl moiety of malathion is bound to the serine residue in AChE^1^. The formation of HSA adducts with various substances, such as 1,2-benzoquinone and ethylene oxide is also known^13^. Using LC-Q/TOF–MS, we found that malathion formed adducts with K and CP residues in HSA, namely, malathion was divided into two moieties: the phosphorothionyl moiety, which is bound to an ε-amino group of K residue, and the alkylthiol moiety, which is bound to a thiol group of CP residue. To our knowledge, this is the first observation that malathion was bound to amino acids other than serine. Because only one CP residue is found in the HSA sequence, we conclude that malathion is specifically bound to ^34^Cys in HSA. Contrary to the CP residue, there are multiple K residues in the HSA sequence. A previous report showed that organophosphorus compounds were preferably bound to several K residues of HSA^10^. We intend to clarify whether malathion binds to a specific K residue in HSA or not in our future study.

Malathion concentration in blood is one important factor for determining malathion-poisoning death. The K- and CP-adducts were specifically detected by LC-Q/TOF–MS, and their quantification was well validated. Lethal malathion concentration in blood is higher than 0.6 μg/mL^3^. This indicates that the detection of K- or CP-adduct, instead of malathion per se, can be used to determine death by malathion poisoning. In other words, both K-adduct and CP-adduct could be used as a biomarker of malathion poisoning, and the ingested amount of malathion could be calculated from the concentrations of the K-adduct and the CP-adduct. Recently, we found that the ingested amount of methomyl, another type of pesticide, could be calculated from the concentration of a specific adduct in blood protein^11^.

Although measuring the inhibition of choline esterase activity in blood of autopsied subject is effective to determine malathion poisoning, a more precise method for the quantification of malathion concentration to unambiguously determine the cause of death by malathion poisoning is desired. In this study, blood malathion concentration was lower than the lethal concentration of malathion (Table 4). Malathion concentration (9.5 μg/mL) calculated from the amount of K-adduct was reasonable as the lethal concentration of malathion. This proved that the K-adduct could be used as a biomarker of malathion poisoning in clinical practice. The CP-adduct was not detected in blood sampled from autopsied subject who died of intentional malathion poisoning, although quantification of the CP-adduct was also validated in vitro, as in the K-adduct. As shown in Fig. 5, the CP-adduct was easily and completely decomposed by GSH. As GSH and/or other reductants are present in abundance in blood, particularly, red blood cells, the CP-adduct was not detected in blood sampled from autopsied subject who died of intentional malathion poisoning. Therefore, only the K-adduct is useful in clinical practice.

In conclusion, the detection of intact malathion in post-mortem blood was difficult because it formed adducts with amino acid residues in HSA, such as lysine (K) and cysteinylproline (CP). Because only the K-adduct was detected in blood of autopsied subject who died of intentional malathion ingestion, the K-adduct could be used as a biomarker of malathion poisoning. In addition, we were able to calculate blood malathion concentration on the basis of the amount of K-adduct in blood. We expect that other organophosphoryl pesticides having structures in common with malathion would also produce similar adducts in HSA.

## Supplementary Information

Supplementary Information.
