# Supplementary material for: Effects of human serum albumin on post-mortem changes of malathion
Source: Sci Rep. 2021 Jun 2;11:11573. doi: 10.1038/s41598-021-91145-y (PMC8172840; doi:10.1038/s41598-021-91145-y)

Supplementary Information

Yoshikazu Yamagishi^1,2^, Hirotaro Iwase^2^, and Yasumitsu Ogra^*,1,2^

^†^*Laboratory of Toxicology and Environmental Health, Graduate School of Pharmaceutical Sciences, Chiba University, Chuo, Chiba 260-8675, Japan*

^‡^*Department of Legal Medicine, Graduate School of Medicine, Chiba University,* *Chuo, Chiba 260-8675, Japan*

*Corresponding Author

Yasumitsu Ogra

Laboratory of Toxicology and Environmental Health, Graduate School of Pharmaceutical Sciences, Chiba University

Tel: 81-43-226-2944 Fax: 81-43-226-2944

E-mail: ogra@chiba-u.jp

**Figure legends**

Fig. S1 Elution profiles at *m/z* 303.0120 in solution.

One μg/mL malathion was incubated without (a and b) and with HSA (c and d) or Hb (e and f). Incubation times were 0 (a, c, and e) and 24 hr (b, d, and f).

Fig. S2 Relationship between malathion concentrations and peak areas of K-adduct (a) and CP-adduct (b).

**Fig. S1**


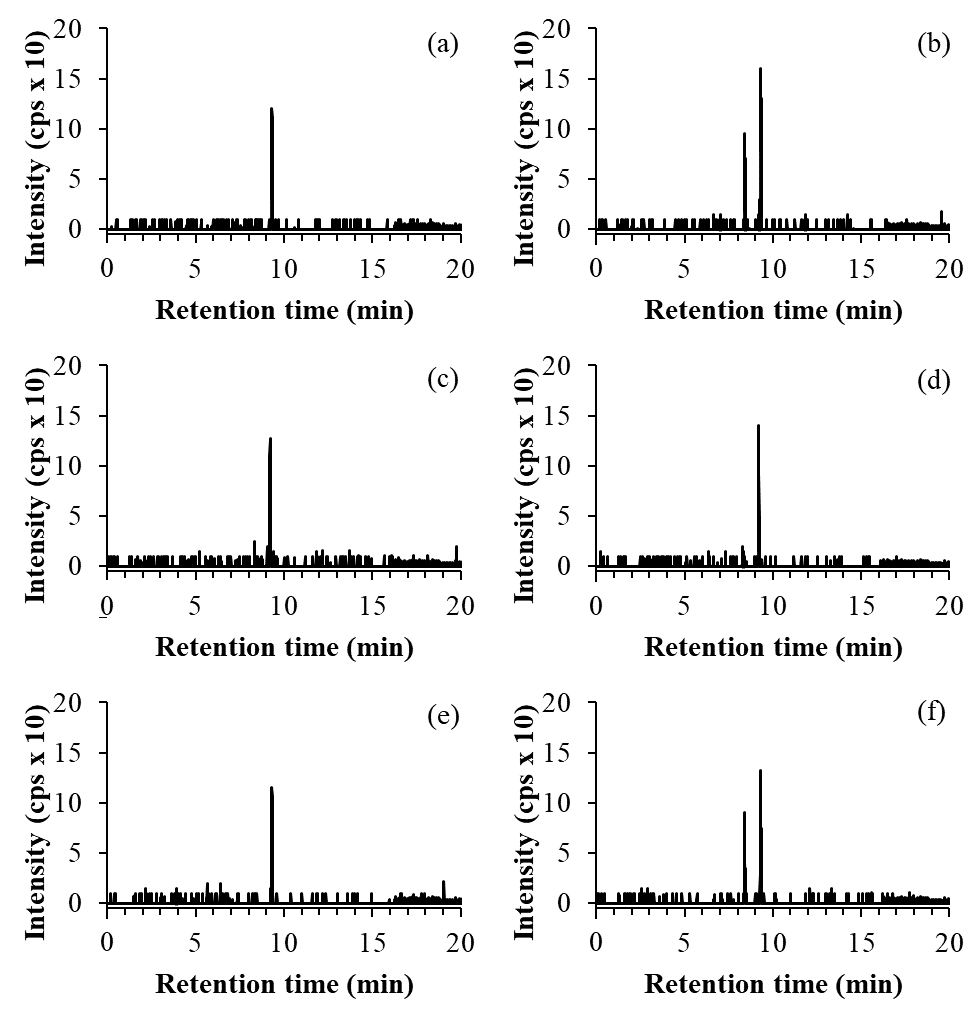


**Fig. S2**


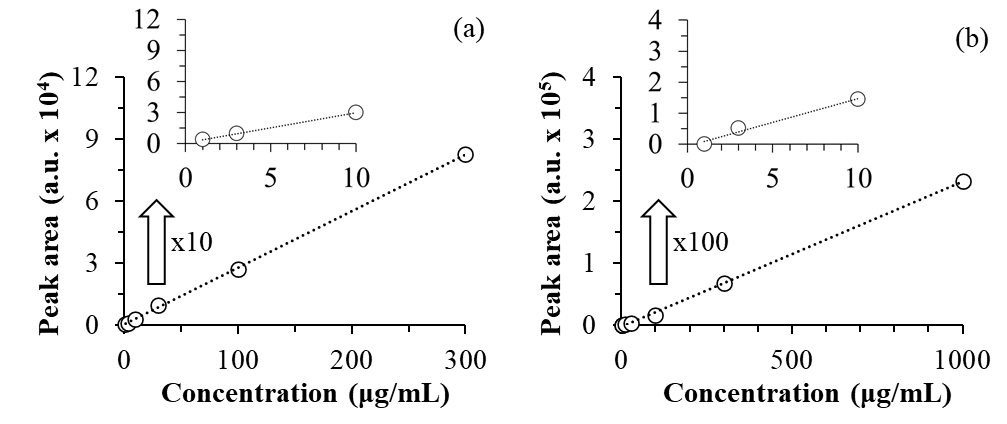

Supplement: Supplementary file 1 — Supplementary Information. [file 41598_2021_91145_MOESM1_ESM.docx]
